# The Role of Insulin Resistance/Hyperinsulinism on the Rising Trend of Thyroid and Adrenal Nodular Disease in the Current Environment

**DOI:** 10.3390/jcm7030037

**Published:** 2018-02-26

**Authors:** Agathocles Tsatsoulis

**Affiliations:** Department of Endocrinology, School of Health Sciences, University of Ioannina, 45110 Ioannina, Greece; atsatsou@uoi.gr; Tel.: +30-695-178-0072

**Keywords:** thyroid nodules, adrenal incidentalomas, insulin resistance, hyperinsulinemia, mitogenic effects, metabolic syndrome

## Abstract

Thyroid follicular cells, as well as adrenocortical cells, are endowed by an intrinsic heterogeneity regarding their growth potential, in response to various stimuli. This heterogeneity appears to constitute the underlying cause for the focal cell hyperplasia and eventually the formation of thyroid and adrenal nodules, under the influence of growth stimulatory factors. Among the main stimulatory factors are the pituitary tropic hormones, thyroid-stimulating hormone (TSH) or thyrotropin and adrenocorticotropic hormone (ACTH), which regulate the growth and function of their respective target cells, and the insulin/insulin-like growth factor system, that, through its mitogenic effects, can stimulate the proliferation of these cells. The predominance of one or the other of these growth stimulatory factors appears to determine the natural history of thyroid and adrenal nodular disease. Thus, iodine deficiency was, in the past, the main pathogenic factor responsible, through a transient rise in TSH secretion, for the endemic nodular goiter with the characteristic colloid thyroid nodules among the inhabitants in iodine deficient areas. The correction of iodine deficiency was followed by the elimination of endemic colloid goiter and the emergence of thyroid autoimmunity. The recent epidemic of obesity and metabolic syndrome (MS), or insulin resistance syndrome, has been associated with the re-emergence of nodular thyroid disease. A parallel rise in the incidence of benign, nonfunctional adrenocortical tumors, known as adrenal incidentalomas, has also been reported in association with the manifestations of the MS. It is likely that the compensatory to insulin resistance hyperinsulinemia may be responsible for the rising trend of thyroid and adrenal nodular disease in the current environment.

## 1. Introduction

Thyroid and adrenal nodular disease is defined by the presence of solitary or multiple nodules in the parenchyma of these glands, that is not of malignant or inflammatory etiology. The process of nodular formation appears to arise from an underlying intrinsic heterogeneity of the endocrine cells in responding to various growth stimulatory factors, and this propensity is transferred from the mother cells to their progeny [1,2,3,4,5].

The main thyroid and adrenal cell growth stimulatory factors are the tropic hormones, thyroid stimulating hormone (TSH) and adrenocorticotropic hormone (ACTH), which are known to stimulate both proliferation and function of their respective target cells. In addition, the insulin/insulin-like growth factor (IGF) system, through its mitogenic effects, appears to stimulate the growth of these endocrine cells [6,7,8].

**Figure 1:**
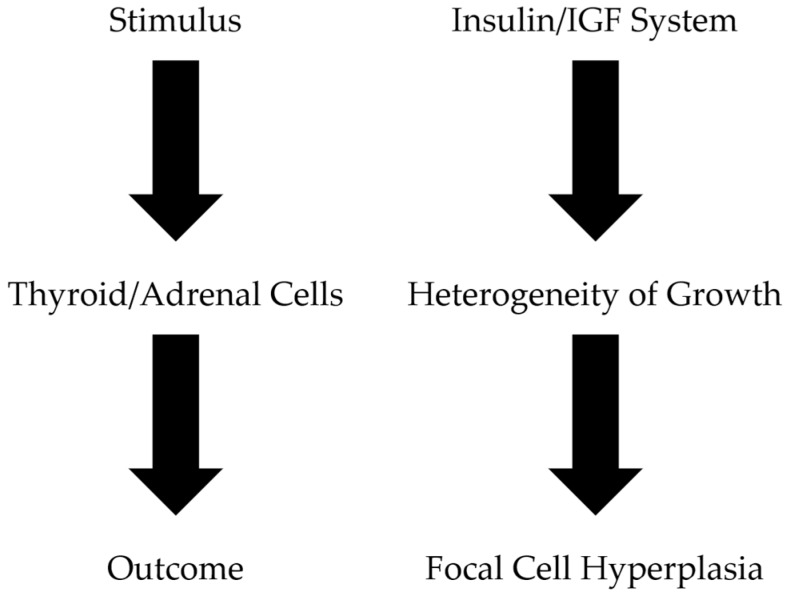
Pathogenesis of thyroid/adrenal nodule formation. The primary cause of the focal cell hyperplasia, characteristic of the thyroid and adrenal nodular disease, appears to be the intrinsic heterogeneity of target cells in responding to growth stimulating factors. IGF: insulin-like growth factor.

The natural heterogeneity in growth and function, which characterizes the thyroid follicular cells, constitutes the primary underlying cause for the focal thyroid cell hyperplasia, leading to the clinical development of nodular thyroid disease. It is likely that the same mechanism is responsible for the development of adrenal nodular disease. On the other hand, the various factors, that stimulate the cells to proliferate and differentiate, appear to determine the evolution and the natural history of thyroid and adrenal nodular disease [2,4,5] (Figure 1).

The recent epidemic of obesity and metabolic syndrome, has been accompanied by an increase in the incidence of hyperplastic thyroid nodules, as well as benign, non-functional adrenocortical tumors, also known as adrenal incidentalomas (detected incidentally by various radiological methods in asymptomatic individuals) [9,10].

In the present review, evidence for the rising prevalence of thyroid nodules and adrenal incidentalomas, in association with the manifestations of the Metabolic Syndrome (MS), is provided and the possible role of the compensatory to insulin resistance hyperinsulinemia for this trend is discussed.

## 2. The Rising Prevalence of Thyroid and Adrenal Nodular Disease in the Current Environment

In the past, iodine deficiency was the main cause of endemic goiter in iodine deficient areas of the world. In response to reduced iodine supply and the associated transient increase in TSH secretion, the thyroid gland undergoes a period of focal thyroid cell hyperplasia, but eventually, because of iodine repletion or the decreased requirement for thyroid hormone, the thyroid enters a resting phase characterized by colloid storage and the formation of colloid nodular goiter [2,4,11].

The implementation of iodine prophylaxis programs or, in some countries, the “silent iodine prophylaxis” due to improvement in socioeconomic conditions, was followed by a gradual elimination of endemic goiter. However, the transition from iodine deficiency to sufficient or excess iodine has been associated with the emergence of thyroid autoimmunity [12,13,14].

In recent years, the epidemic of obesity and the metabolic/insulin resistance syndrome, as a result of the current lifestyle, has been accompanied by the re-emergence of thyroid nodular disease in the form of nodular hyperplasia [15]. On the other hand, increasing evidence also suggests that the prevalence of non-functional adrenal tumors is also on the rise, in parallel with the increasing incidence of the MS/insulin resistance syndrome [16]. Moreover, a relationship between thyroid nodules and non-functioning adrenal incidentalomas was detected, in association with the MS [10].

It appears, therefore, that thyroid nodules and adrenal incidentalomas may coexist in subjects with insulin resistance and compensatory hyperinsulinemia, and may represent another manifestation of the metabolic syndrome.

## 3. Metabolic/Insulin Resistance Syndrome and Compensatory Hyperinsulinism—Mitogenic Effects

MS or insulin resistance syndrome is characterized by the presence of abdominal obesity, hypertension and/or dyslipidemia, linked to insulin resistance, and carries an increased risk for developing type 2 diabetes and cardiovascular disease [17].

Insulin, an anabolic hormone, is mainly secreted after food intake and is responsible for the storage of the absorbed energy in the form of glycogen in liver and triglycerides in adipose tissue. Apart from these metabolic effects, insulin is also known to stimulate replication of cells and exert anti apoptotic effects, leading to cellular proliferation and tissue hyperplasia [18].

It is believed that resistance of target tissues to the metabolic effects of insulin is an adaptive mechanism, activated whenever the organism needs an increased supply of energy, in the form of glucose, to the brain. In cases, such as acute stressful events or whenever the organism is fighting off an infection, the anabolic effects of insulin are temporarily blocked by the action of stress hormones or proinflammatory cytokines. This facilitates the mobilization of stored energy to be used for “a fight or flight” response or to fight off the infection [18,19].

The current way of life, characterized by high calorie intake, low physical activity and chronic stress, favors the activation of the same mechanism of insulin resistance in individuals with a tendency for central fat distribution, on a chronic basis. Thus, storage of surplus energy, in the form of triglycerides, in abdominal fat depots, that far exceeds their storage capacity leads to adipocyte hypertrophy and dysfunction with resultant systemic low-grade inflammation and lipid overflow to peripheral tissues. In turn, the accumulation of lipid metabolites in liver and muscle cells, together with the inflammatory factors and the stress hormones activate the mechanism of insulin resistance on these insulin target cells, leading to the metabolic manifestation of the MS [18,19].

The ensuing insulin resistance puts a strain on pancreatic β-cells, which keep secreting excessive amounts of insulin in order to maintain normal glucose homeostasis. It should be noted here that the resistance of target cells to insulin involves only its metabolic actions and not the mitogenic effects, which are mediated by a different intracellular pathway [17,18].

This compensatory hyperinsulinism appears to be responsible for the mitogenic manifestations of the MS, including acanthosis nigricans, skin tags (acrochordons), and hyperplasia of various tissues and organs [18].

## 4. Association of Insulin Resistance/Hyperinsulinism with Increased Risk of Thyroid and Adrenal Nodular Disease—Clinical Evidence

Accumulating evidence suggest that the recent increase in the incidence of thyroid nodular disease, in iodine replete areas, is related to the increased prevalence of obesity and insulin resistance. Thus, several recent studies have shown an association of insulin resistance with thyroid nodular disease [15].

These were mainly cross-sectional studies designed to evaluate either the prevalence of insulin resistance in subjects with benign thyroid nodules as compared to controls, or, alternatively, the prevalence of thyroid nodules in subjects with insulin resistance, as evidenced by the measurement of Homeostatic Model Assessment for Insulin Resistance (HOMA IR) or the presence of MS/type-2 diabetes and in subjects with skin tags, a clinical sign of hyperinsulinism.

The results of the first type of studies have shown that in subjects with thyroid nodules there is associated insulin resistance and hyperinsulinemia. Consistent with these findings, patients with nodular disease were found to have higher HOMA-IR values when compared with individuals with a normal thyroid gland [20,21,22,23]. A correlation was found between the nodular size and HOMA-IR levels [21]. On the other hand, it was shown that, due to metabolic syndrome, patients with hyperinsulinemia have increased thyroid volume and nodule prevalence. A similar study showed that patients with skin tags, as mark of hyperinsulinemia, have a higher prevalence of ultrasound-detected thyroid nodules and larger thyroid glands [24,25]. Finally, a relationship was found between obesity and hyperinsulinemia with increased susceptibility for thyroid nodules in a pediatric population [26]. In addition to TSH, insulin is also a thyroid growth stimulatory factor, which may cause an increase in thyroid volume and the formation of hyperplastic thyroid nodules. Evidence also suggests that IGF-1-dependent and TSH-independent signaling may play a role in growth regulation of the human thyroid gland. This is supported by conditions not associated with increased TSH secretion, like acromegaly, in which high levels of intra-thyroidal IGF-1 may contribute to thyroid nodular formation [27].

Thus, the likely culprit for the above association appears to be the hyperinsulinemia that accompanies the insulin resistance syndrome.

There is also evidence for an association of insulin resistance/hyperinsulinemia with adrenocortical tumor formation. This evidence comes from case-control studies and is supported by in-vitro findings. Thus, an increasing number of recent studies have reported that insulin resistance, as assessed by HOMA IR index, and several of the features of the MS, including central adiposity, hypertension, dyslipidemia, and type 2 diabetes as well as cardiovascular risk factors, occur with higher prevalence in patients with non-functioning adrenal incidentalomas, as compared to age-matched healthy subjects [28,29,30,31]. Furthermore, a correlation between the size of adrenal incidentalomas with insulin resistance was reported [32].

The above cross-sectional studies indicate an association of insulin resistance with adrenal incidentalomas [33]. However, they cannot determine which one comes first [34]. Although it remains unclear whether the adrenal tumors are developing from a primary insulin resistance and compensatory hyperinsulinemia or whether insulin resistance is secondary to the slight hyper secretion of cortisol by the adrenal mass, one has to rely on the interpretation of the currently available data.

Clinical evidence may suggest a causal role of insulin resistance and primary hyperinsulinemia on adrenal tumor growth. This hypothesis was first raised by a study in which the presence of insulin resistance/hyperinsulinemia in patients with adrenal incidentalomas was investigated [16]. By performing a 2 h 75 g oral glucose tolerance test (OGTT), and measuring both glucose and insulin, the authors found that all of the patients were insulin resistant. Furthermore, they also found that insulin was clearly able to stimulate in vitro the proliferation of NCI-H295R cells in a time and dose dependent manner [16]. In another study, after performing an OGTT and a hyperinsulinemic euglycemic clamp, the authors found that patients with nonfunctioning adenomas were more insulin-resistant than a matched control group and that insulin resistance was related to the tumor size [35]. It was concluded that hyperinsulinemia may play an important role in adrenal tumor growth.

On the other hand, hyperinsulinemia associated with apparently nonfunctioning adrenal tumors may be a consequence of a slight and often undetectable cortisol hyper secretion by the adrenal tumor [36]. Cortisol is known to induce insulin resistance, as well as impairment of lipid profile and blood pressure. Cortisol hypersecretion in non-functioning adrenocortical tumors is often undetectable with routine diagnostic assays. An increase of usually undetected cortisol metabolism markers has been reported in patients with apparently nonfunctioning adrenal adenomas as compared to a control group [37]. However, these results are not supported by a recent study which reported no difference in the urinary excretion of cortisol metabolites between subjects with non-functioning adrenal adenomas and controls [38].

Additional studies have reported a decrease of insulin resistance as well as an improvement of diabetes and blood pressure after removal of non-functioning adenomas [39,40]. In contrast, other studies did not detect any metabolic improvement, after surgery in non-functioning adrenocortical tumors [41].

Notwithstanding these considerations, the weight of evidence from the recent clinical data favors a causal role of hyperinsulinemia in determining the development of adrenal tumors. This view is further supported by the finding that insulin may stimulate in-vitro the growth of an adrenal cell line [16], as well as by the observation that the degree of insulin resistance was directly correlated to the size of the adrenal mass [35]. Taken together, these findings indicate that the hyperinsulinemia, which accompanies the insulin resistance, could be mitogenic on the adrenal cortex by inducing the activation of the insulin/IGF receptors.

## 5. The Insulin/IGF System and Its Involvement in Thyroid and Adrenal Cell Hyperplasia

As mentioned earlier, insulin exerts both metabolic and mitogenic effects to target cells. In patients with MS and insulin resistance, the later applies only to the metabolic effects of insulin, whereas the mitogenic action is maintained and even exaggerated by the associated hyperinsulinemia [18].

The insulin/IGF system consists of three ligands (insulin, IGF-1, and IGF-2), three tyrosine kinase receptors (the insulin receptor, the IGF-1 receptor and the mannose-6 phosphate IGF-2 receptor), and six IGF-binding proteins (IGFBP-1 to -6), which regulate the half-life and the biological activity of IGFs, while insulin circulates in free form. The insulin receptor (IR)and IGF-1R have homologous structures and can heterodimerize leading to the formation of insulin/IGF-1 hybrid receptor. The human IR exists in two isoforms (IR-A and IR-B), generated by alternative splicing of the IR gene with the exclusion (IR-A) or inclusion (IR-B) of 12 amino acids encoded by exon 11. Insulin and IGFs bind with different affinity to IR and IGF-1R [42,43].

After ligand binding, phosphorylated receptors activate two main signaling pathways, the PI3K signaling pathway, which mediates the metabolic actions of insulin, and the mitogen-activated protein kinase (MAPK) cascade, which is involved in the regulation of cellular proliferation and gene expression [18]. As was mentioned earlier, it is the metabolic pathway that is affected in the insulin-resistant individuals, whereas the mitogenic pathway remains intact.

The IGF system plays an important role in regulating normal development and growth of the thyroid and appears to be involved in thyroid cell hyperplasia [7]. Functional IR and IGF-1R are expressed in both cultured thyroid cells and tissue specimens, and also adenoma cell lines may synthesize IGF-1, which stimulates cell growth in an autocrine manner [44].

On the other hand, all of the components of the insulin/IGF system are expressed in human fetal adrenals. In normal adrenal glands, insulin receptors as well as IGF-1 and IGF-2 receptors are expressed in adrenal cortex and may play a role in the development and growth of the adrenal glands [8,45].

In patients with insulin resistance, the hyperinsulinemia may enhance the bioavailability of IGF-1 and IGF-2 by inhibiting the production of the IGFBP-1 and IGFBP-2 in the liver [46]. The increased bioavailability of IGFs may contribute to thyroid cell proliferation acting through the IGF-1R or the IR itself. Yet, hyperinsulinemia, by directly activating the IR-A, may favor its mitogenic actions through the induction of the pro-mitogenic MAPK cascade [18].

**Figure 2:**
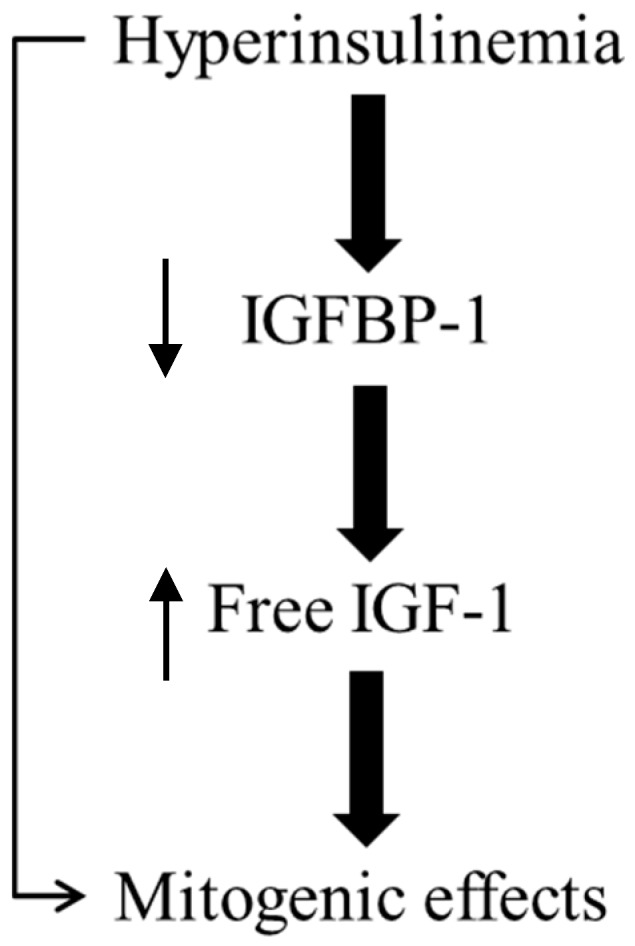
Insulin resistance/hyperinsulinism mitogenic effects. The hyperinsulinemia compensatory to insulin resistance, appears to exert its mitogenic effects directly by acting on its own receptors or indirectly, by reducing the production of IGFBP-1 in the liver and thus increasing the bioavailability of free IGF-1. IGFBP-1: insulin-like growth factor-binding protein 1.

With the above in mind, it is suggested that, in patients with insulin resistance, the compensatory hyperinsulinemia with the concomitant increased activity of the IGF axis may explain the increase in the prevalence of thyroid and adrenal nodular disease (Figure 2).

## 6. Conclusions and Future Perspectives

Recent evidence suggests a role of insulin resistance/hyperinsulinism in the evolution of thyroid nodular disease. This involves the transition from endemic colloid goiter, due to iodine deficiency and the associated transient increase in TSH secretion, to the increase in the incidence of nodular thyroid hyperplasia, due to hyperinsulinemia, as a manifestation of the current epidemic of the insulin resistance syndrome. In the interim, the elimination of iodine deficiency was followed by the disappearance of endemic nodular goiter and the emergence of thyroid autoimmunity, as a result of the increase in iodine intake.

The changing phenotype of nodular thyroid disease, i.e., from colloid to hyperplastic nodular goiter, is mainly due to changes in the thyroid stimulatory factors acting on thyroid cells, that are characterized by an intrinsic heterogeneity regarding their growth potential and function. Thus, in the case of colloid goiter, the stimulus is the transient increase in TSH secretion as a result of iodine deficiency. The initial rise in TSH appears to be responsible for the focal thyroid hyperplasia. This is followed by a resting phase that is characterized by colloid storage, resulting in the formation of colloid nodules. On the other hand, the hyperinsulinemia constitutes a mild but continuous stimulus for thyroid cell proliferation, leading to increase in thyroid gland volume and the formation of hyperplastic nodules.

Evidence also supports a role for the mitogenic effects of the compensatory to insulin resistance hyperinsulinemia in the formation of non-functional adrenal tumors, and this may explain their rising prevalence along with the manifestations of the MS.

This view opens up a new perspective for the prevention and therapeutic intervention of these common nodular hyperplastic diseases, by focusing on the amelioration of the underlying insulin resistance and the associated hyperinsulinemia. This may be achieved by simple measures of changing our current lifestyle and/or the use of insulin sensitizers. There is preliminary evidence that the use of Metformin may be effective in achieving this goal [46].

It is concluded that the current increase in the incidence of thyroid and adrenal nodular disease in humans is another manifestation of the metabolic/insulin resistance syndrome. It is likely that the compensatory hyperinsulinemia, through its mitogenic effects, is responsible for the re-emergence of nodular thyroid disease and the rising trend of adrenal incidentalomas in the current environment. It is also likely that by tackling the underlying cause of insulin resistance, we may be able to prevent or to treat these hyperplastic endocrine manifestations of the MS.
